# An examination of neurocognition and theory of mind as predictors of engagement with a tailored digital therapeutic in persons with serious mental illness

**DOI:** 10.1016/j.scog.2022.100236

**Published:** 2022-01-17

**Authors:** Tate F. Halverson, Julia Browne, Samantha M. Thomas, Paige Palenski, Roger Vilardaga

**Affiliations:** a Durham VA Health Care System, Durham, NC, United States of America; b Veterans Affairs Mid-Atlantic Mental Illness Research, Education and Clinical Center, United States of America; c Geriatric Research, Education, and Clinical Center, Durham VA Health Care System, Durham, NC, United States of America; d Duke Cancer Institute, Duke University, Durham, NC, United States of America; e Department of Biostatistics and Bioinformatics, Duke University, Durham, NC, United States of America; f Department of Psychiatry and Behavioral Neurobiology, The University of Alabama at Birmingham, Birmingham, AL, United States of America; g Department of Psychiatry and Behavioral Sciences, Duke University, Durham, NC, United States of america

**Keywords:** Digital therapeutic, mHealth, Serious mental illness, Cigarette, Tobacco, Cognition

## Abstract

There is an increasing interest in the development and implementation of digital therapeutics (apps) in individuals with serious mental illness (SMI). However, there is limited understanding of the role of neurocognition and social cognition on engagement with apps. The present study is a secondary analysis of a pilot randomized controlled trial (*N* = 62) comparing a tailored digital intervention to treat tobacco use disorder in individuals with SMI to a standard of care digital intervention for the general population. The purpose of this study was to examine the impact of neurocognition, social cognition, and clinical characteristics on indices of app engagement in users of the tailored app compared to users of the standard of care app. Correlational analyses demonstrated that individuals with low levels of neurocognition and social cognition engaged more often and for longer duration with the tailored app compared to the standard of care app. In a series of multilevel zero-inflated negative binomial models, assignment to the tailored app remained the most robust predictor of app interactions (Risk Ratio [RR] = 1.72; *p* < .01), duration of app use (RR = 6.47; p < .01), and average length of interaction (RR = 2.70; p < .01), after adjusting for key demographic and clinical characteristics, and two measures of cognition. This is one of the first studies to demonstrate that digital therapeutics can be designed to mitigate the impact of neurocognition and social cognition on device engagement in SMI populations. Recommendations are made to advance the use of new analytic models to uncover patterns of engagement with digital therapeutics.

## 1 Introduction

Digital therapeutics (e.g., smartphone applications [apps]) to treat mental health problems are widely available as stand-alone or complementary tools in clinical care (Noel et al., 2019). With the rapid growth of mental health apps developed to reduce symptoms and increase functioning in common mental health disorders (e.g., anxiety), there is increasing interest in the development and application of apps for individuals with serious mental illness (SMI; Batra et al., 2017; Hatch et al., 2018). SMI includes schizophrenia, bipolar, and recurrent major depressive disorders classified together to reflect the chronicity, impaired functioning, and need for long-term treatment associated with these diagnoses. Integration of apps in SMI treatment may be particularly appealing given barriers to treatment access and engagement such as self-stigma, reliable transportation, symptom severity, and limited community mental health resources (Dixon et al., 2016; Firth et al., 2016; Hatch et al., 2018; Horsselenberg et al., 2016). Moreover, individuals with SMI own smartphones at rates comparable to the general population and qualitative and quantitative findings demonstrate overwhelming support and enthusiasm from individuals with SMI for treatment-focused apps (Beard et al., 2019; Bell and Alvarez-Jimenez, 2019; Carpenter-Song et al., 2020; Depp et al., 2016; Firth et al., 2016; Gowarty et al., 2020; Young et al., 2020).

To this end, apps are increasingly utilized in the treatment of SMI in a variety of ways (e.g., stand-alone interventions, symptom monitoring, adjunctive therapeutic support) with demonstrated feasibility and efficacy (Batra et al., 2017; Ben-Zeev et al., 2018; Bucci et al., 2018; Depp et al., 2016; Eisner et al., 2019; Firth and Torous, 2015; Fortuna et al., 2018; Liu et al., 2019). Yet, there is variable engagement with apps among users with SMI (e.g., a small subset of users often account for a large proportion of app engagement) and the real-world uptake of smartphone apps by clinics and users remains relatively low (Torous et al., 2017; Torous et al., 2018), which is concerning given that app engagement is linked with improved outcomes (Ben-Zeev et al., 2018; Best et al., 2019; Browne et al., 2021; Harvey et al., 2020). Therefore, knowledge of the putative barriers to app engagement in this population is necessary to guide strategies for facilitating adequate uptake.

Due to the limited number of studies, characteristics associated with app engagement in SMI have yet to be consistently identified (Killikelly et al., 2017; Liu et al., 2019). The few existing studies have focused on the role of SMI user demographics and clinical characteristics on engagement. Older age and higher education level predicted better app engagement in one study (Arnold et al., 2019). More severe symptoms predicted better app engagement in some studies but lower engagement in others (Buck et al., 2020; Granholm et al., 2012; Killikelly et al., 2017). Likewise, other studies did not find significant relationships between these same user demographics, clinical characteristics, and app engagement (Killikelly et al., 2017; Luther et al., 2020; Niendam et al., 2018).

Despite the range of neurocognitive (e.g., working memory) and social cognitive (e.g., theory of mind) impairments present in SMI (Green et al., 2015; LeMoult and Gotlib, 2019; Reichenberg, 2010; Vöhringer et al., 2013; Zanelli et al., 2019), cognition as a critical predictor of app engagement has been largely understudied. One of the few studies to investigate the relationship between cognition and app engagement found no significant relationship between neurocognition and engagement with an app designed to monitor symptoms and provide psychoeducation for individuals with bipolar disorders (Bonnín et al., 2021). Results from cognitive training trials identified better baseline neurocognitive performance as a predictor of treatment engagement (Best et al., 2019; Thomas et al., 2018), whereas results from a text-based intervention did not find neurocognition to be a significant predictor of engagement (Luther et al., 2020). It is unclear how these results may generalize to digital therapeutics.

The present study examined neurocognition and social cognition as factors associated with app engagement in individuals with SMI in a pilot randomized controlled trial (RCT; NCT03069482) comparing a smoking cessation app tailored for individuals with SMI, Learn to Quit (LTQ; Vilardaga et al., 2020, Vilardaga et al., 2018), with a smoking cessation app developed by the National Cancer Institute (NCI) for the general population, QuitGuide. In addition to engagement with LTQ or QuitGuide, individuals also received concurrent nicotine replacement therapy. LTQ was designed to address cognitive impairments in this population (Vilardaga et al., 2018). Consequently, neurocognition and theory of mind were directly measured in this trial. This pilot RCT demonstrated greater reduction in cigarettes, more app interactions, and longer durations of app use in the LTQ condition compared with QuitGuide (Vilardaga et al., 2020). This secondary analysis study aimed to evaluate whether treatment condition was significantly associated with app engagement after adjusting for several dimensions of neurocognition, theory of mind, and key baseline and clinical characteristics. It was hypothesized that the tailored LTQ condition would be significantly associated with app engagement after adjusting for other important clinical, demographic, and cognitive variables. Finally, the study descriptively examined patterns of association between these key variables and app engagement, both between and across treatment conditions.

## 2 Methods

All methods and procedures were approved by an Institutional Review Board and all participants provided written informed consent. Detailed information regarding study methods and procedures are described elsewhere (Vilardaga et al., 2020).

### 2.1 Participants

Inclusion criteria were: (a) an ICD-10 diagnosis of SMI (schizophrenia, schizoaffective disorder, bipolar disorder, or recurrent major depressive disorder), (b) self-reported smoking five or more cigarettes per day and a carbon monoxide breath test of more than 6 ppm, (c) desire to quit smoking in the next 30 days, (d) at least 18 years of age, (e) medically eligible to use nicotine replacement therapy, (f) fluent in English, (g) adherent to psychiatric treatment, and (h) living in stable housing. Exclusion criteria were: (a) problematic alcohol or illicit drug use in the last 30 days, (b) acute psychotic episode or unsafe to participate in the study, (c) pregnant or the intention to become pregnant, or (d) currently receiving smoking cessation treatment. All inclusion criteria, including completion of the carbon monoxide breath test, were established during an assessment appointment completed before study randomization.

### 2.2 Interventions

All participants (*N* = 62) received combined nicotine replacement therapy (C-NRT; transdermal patch and lozenges) for 8 weeks with monitoring provided by a study physician. In addition to C-NRT, participants were randomized to LTQ (*n* = 33) or QuitGuide (*n* = 29).

LTQ is a theory-based smoking cessation app designed for persons with SMI based on principles of Acceptance and Commitment Therapy, US Clinical Practice Guidelines, and psychoeducation on NRT. LTQ is comprised of 28 modules that facilitate learning of smoking cessation content and skills, as well as daily check-ins of mood, smoking urges, and cigarette use. LTQ was developed according to user-centered design best practices which involved a multi-phase development process incorporating feedback from individuals with SMI and their providers (e.g., emphasis on visual content to convey information, use of storytelling with short sentences, reinforcing small victories towards quitting smoking) along with evidence-based smoking cessation content (Vilardaga et al., 2018). To specifically address cognitive impairment, LTQ emphasized design elements such as simple screens, large buttons, simple app structure, and inclusion of simple cartoon vignettes without audio or moving video to reduce cognitive load. Users were also able to control the speed of content presentation and stop or review vignettes for as long as needed. Behavioral principles (i.e., successive approximations, multiple exemplar training) were used to enhance comprehension and retention by gradually presenting increasingly complex content, and by presenting the same concepts and skills via a wide range of examples and demonstrations repeated across multiple modules.

QuitGuide is a publicly available app (www.smokefree.gov) developed by NCI for the general population (i.e., not specifically for individuals with comorbid psychiatric symptoms) that provides psychoeducation, a tool for tracking smoking behavior, and strategies for quitting smoking. Similar to LTQ, QuitGuide provides evidence-based smoking cessation content and skills, as well as daily check-ins of mood, smoking urges, and cigarette use. Unlike LTQ, QuitGuide presents smoking cessation content and psychoeducation in a more straightforward and less dynamic way, similar to receiving a brochure (e.g., content is not presented in cartoon vignettes, principles of successive approximation and multiple exemplar training not emphasized).

### 2.3 Measures

#### 2.3.1 Engagement

Engagement was measured using Google Analytics and operationalized in three ways: (1) total app interactions per day, (2) total minutes of app use per day, and (3) average duration of interaction per day. Interactions capture app-specific actions (i.e., actively clicking on content such as a check-in or reviewing psychoeducation materials) that were pre-specified. Minutes per day of app use and average length of app interaction reflect duration of app use.

#### 2.3.2 Neurocognition and social cognition

Neurocognition was measured with the Brief Assessment of Cognition in Schizophrenia (BACS; Keefe et al., 2004), a battery of neurocognition tests which assess verbal memory, working memory, motor speed, attention, executive function, and verbal fluency. The BACS was developed for use in schizophrenia disorders but has also been used to assess neurocognition in SMI more broadly including depression and bipolar disorders (Chih-Ken et al., 2018; Huang et al., 2020). Individual domain and total composite scores were converted to standardized *Z*-scores according to participant age and sex based on available normative data. Social cognition was measured with the False Belief Task (Langdon and Coltheart, 1999) total score, which assesses theory of mind.

#### 2.3.3 Clinical characteristics

Symptoms were assessed with the Brief Symptom Inventory (BSI; Derogatis, 1992) and the Positive and Negative Syndrome Scale (PANSS; Kay et al., 1987). The BSI is a self-report measure that yields a global severity index of distress with subscale scores measuring anxiety and depression. The PANSS is a clinical interview that measures positive, negative, and general symptoms. The Avoidance and Inflexibility Scale (AIS; Farris et al., 2015) was administered as a smoking-specific measure of experiential avoidance, unwillingness to experience aversive internal experiences, with higher scores reflecting more experiential avoidance.

### 2.4 Procedures

Eligible participants were randomized 1:1 to an app condition (LTQ or QuitGuide) stratified by diagnosis (psychotic or mood disorder). Smartphones were provided to participants along with up to four sessions of smartphone coaching from the study team. Assessments were administered at baseline, 4 weeks, 8 weeks, 12 weeks, and 16 weeks with the exception of the BACS, which was only administered at baseline. If participants completed all measures, they earned $110 and retained their smartphone at the completion of the trial.

### 2.5 Data analysis

**Fig. 1:**
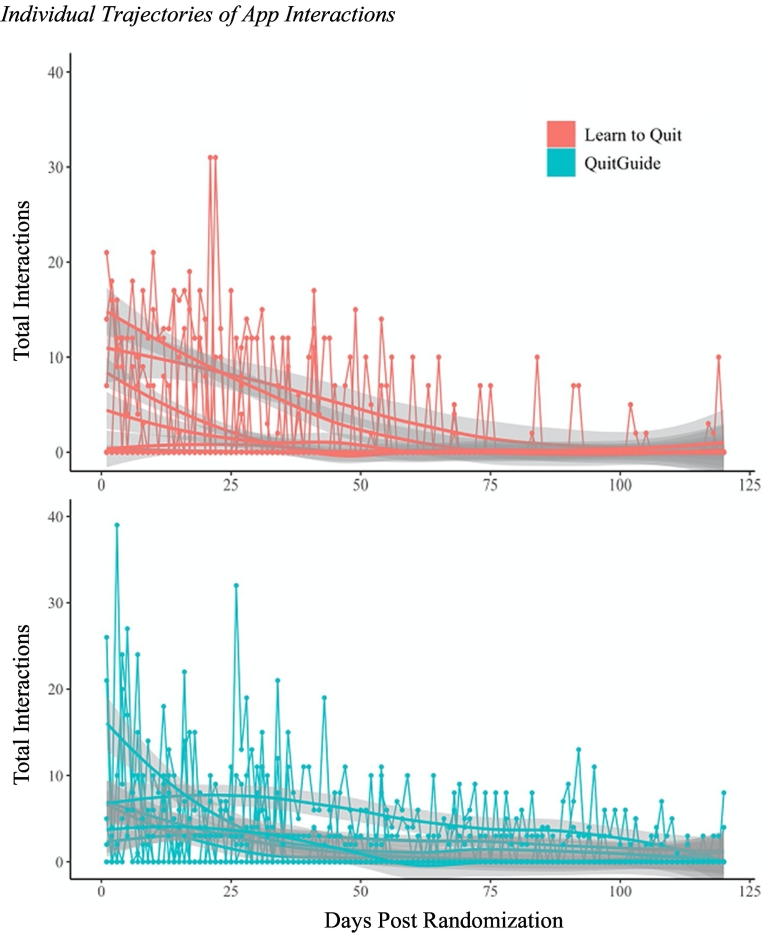

All statistical analyses were conducted in *R* using the glmmADMB package (Bolker et al., 2012). Due to a software update, app interaction duration was not available for eight participants in the QuitGuide group. Analyses suggested that this software update was unrelated to participant demographics and that data were Missing Completely At Random (MCAR; all *X*^2^ tests *p* > .05). Considering that app interaction duration is a dependent variable of interest and MCAR pattern of missingness, these participants were excluded from analyses when app duration (i.e., total duration, average interaction) was the dependent variable following guidelines for handling missing data in RCTs (Jakobsen et al., 2017). Treatment groups were compared on demographics, clinical characteristics, and cognitive performance using *t*-tests (continuous variables) and chi-square tests (categorical variables). Bivariate correlations (Pearson for continuous relationships, Spearman for relationships with categorical variables), adjusted for multiple comparisons using Holm-Bonferroni correction, were estimated among engagement metrics and potential predictors of app engagement (i.e., demographics, clinical characteristics, cognitive performance). Given interindividual variability present in app engagement (e.g., Torous et al., 2017), outcomes of interest were inspected visually per participant (Fig. 1). To account for interindividual variability, multilevel models were estimated with two error terms (u_*oj*_ for between individual observations and r_*ij*_ for between individual observations nested within participants over time). Dependent variables were daily indices of app engagement (i.e., number of app interactions, minutes of use, average duration of interaction). The distribution of app engagement outcomes was negatively skewed with a large proportion of zeros (Supplementary Fig. 1). To appropriately model the data, multilevel zero-inflated negative binomial models (Diallo et al., 2017) were used to analyze the effect of app engagement predictors on each engagement outcome. Unadjusted models were first conducted to analyze the effect of treatment alone and then each model was adjusted for treatment, demographic, clinical, and cognitive variables. Due to high correlations among BACS subtests, the BACS composite score was used to capture neurocognition performance rather than individual BACS subtests. The following is an example of the adjusted models applied using number of daily interactions where *i* indexes individuals and *j* indexes observations.

$$\text{Model}:{\mathrm{Total Interactions}}_{\mathrm{ij}}=b_{0}+b_{1}\mathrm{Treatment}\;\mathrm{Arm}+b_{2}\mathrm{Age}+b_{3}\mathrm{Sex}+b_{4}\mathrm{Race}+b_{5}\mathrm{Diagnosis}+b_{6}\mathrm{Illness Duration}+b_{7}\mathrm{PANSS Total}+b_{8}\mathrm{BSI}\;\mathrm{Global Severity}+b_{9}\mathrm{AIS}\;\mathrm{Avoidance}+b_{10}\mathrm{BACS Composite}+b_{11}\mathrm{False Belief Task}+\mu \mu_{\text{0j}}+r_{\mathrm{ij}}$$

## 3 Results

### 3.1 Demographics and clinical characteristics

Overall, minimal differences in demographics, clinical characteristics, and cognitive performance were observed between treatment groups (Table 1). LTQ participants had more interactions, longer duration of interactions, and longer average interactions compared with QuitGuide participants.

**Table 1:** Demographic, clinical, and engagement characteristics.

	Learn to Quit *n* = 33	QuitGuide *n* = 29	*p* Values
Demographics			
Age, years	48.4 ± 11.2	45.6 ± 10.9	.32
Male % (*n*)	36.4 (*12*)	44.8 (*13*)	.50

Education level %, (*n*)			
Some/completed high school	33.3 (*11*)	34.5 (*10*)	.92
Some college	27.3 (*9*)	27.6 (*8*)	.98
Associate degree	12.1 (*4*)	17.2 (*5*)	.57
Bachelor's degree or higher	27.3 (*9*)	20.7 (*6*)	.55

Race % (*n*)^a^			
White	48.5 (*16*)	55.2 (*16*)	.60
Black/African American	42.4 (*14*)	37.9 (*11*)	.72
Asian	3.0 (*1*)	0.0 (*0*)	.34
American Indian/Alaskan Native	0.0 (*0*)	3.4 (*1*)	.28
Multiracial	6.1 (*2*)	3.4 (*1*)	.63

Clinical characteristics			
Primary diagnosis % (*n*)			
Schizophrenia Spectrum Disorder	30.3 (*10*)	17.2 (*5*)	.23
Bipolar Disorder	45.5 (*15*)	51.7 (*15*)	.62
Major Depressive Disorder	24.2 (*8*)	31.0 (*9*)	.55
Duration of treatment, years	21.8 ± 14.5	18.6 ± 10.9	.34

PANSS			
Total	52.7 ± 11.0	49.2 ± 9.5	.18
Positive	11.9 ± 3.5	10.1 ± 2.8	.04
Negative	10.8 ± 3.6	10.0 ± 3.7	.38
General	30.0 ± 6.5	29.0 ± 6.5	.55

BSI			
Global severity	1.1 ± 0.7	0.9 ± 0.6	.37
Anxiety	1.2 ± 0.9	0.9 ± 0.7	.17
Depression	1.0 ± 0.8	0.9 ± 0.5	.58
AIS	50.0 ± 9.0	47.8 ± 7.6	.31
Cigarettes per day	21.2 ± 15.5	14.0 ± 6.4	.02
Duration smoking, years	26.0 ± 12.9	26.8 ± 11.3	.78

Cognitive performance			
BACS *Z*-score	−1.0 ± 1.3	−0.9 ± 1.3	.79
False Belief Task	18.1 ± 5.6	16.7 ± 6.0	.36

Engagement metrics			
Total interactions	355.9 ± 315.3	219.3 ± 170.5	.04
Total duration, minutes^b^	254.4 ± 258.9	112.61 ± 103.6	.02
Average interaction, minutes^b^	0.9 ± 0.6	0.5 ± 0.3	<.01

PANSS = Positive and Negative Syndrome Scale, BSI = Brief Symptom Inventory, AIS = Avoidance and Inflexibility Scale, BACS = Brief Assessment of Cognition in Schizophrenia.

a No participants identified as Hispanic so ethnicity is not presented.

b Data missing from 8 participants.

### 3.2 Correlational analysis

Correlations between engagement metrics and potential predictors of app engagement by treatment group are presented in Table 2 (Supplementary Table 1). In the LTQ condition, number of interactions was significantly associated with the BACS subscale *Verbal Fluency – Letter Fluency* (*r* = −.40). Significant associations (*p*s <.01) were observed between duration of app use and the AIS (*r* = .36), and the BACS subscales of *Verbal Memory* (*r* = *−.*40) and *Verbal Fluency – Letter Fluency* (*r* = −.43). Significant associations were also present between average app interaction and age (*r* = .43), BACS *Processing Speed* (*r* = −.44), and the False Belief Task (*r* = .37). No significant associations were observed within the QuitGuide condition.

**Table 2:**
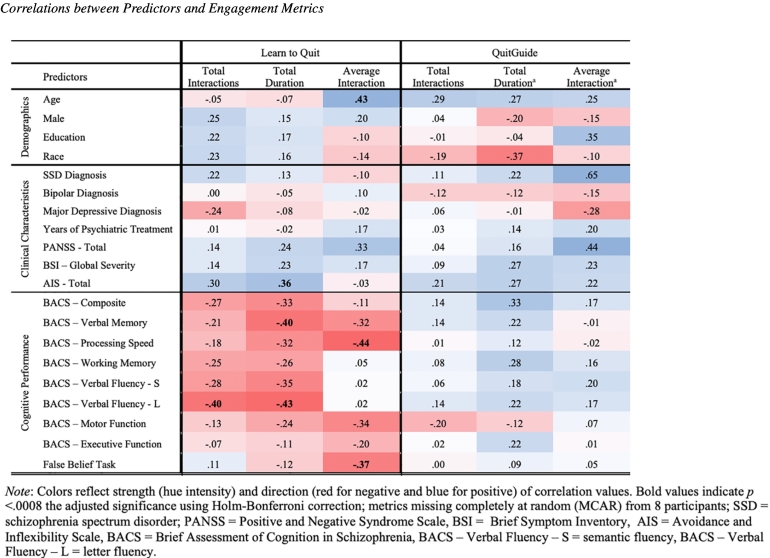
Correlations between predictors and engagement metrics.

*Note*: Colors reflect strength (hue intensity) and direction (red for negative and blue for positive) of correlation values. Bold values indicate *p* < .0008 the adjusted significance using Holm-Bonferroni correction; metrics missing completely at random (MCAR) from 8 participants.

SSD = schizophrenia spectrum disorder; PANSS = Positive and Negative Syndrome Scale, BSI = Brief Symptom Inventory, AIS = Avoidance and Inflexibility Scale, BACS = Brief Assessment of Cognition in Schizophrenia, BACS – Verbal Fluency – S = semantic fluency, BACS – Verbal Fluency – L = letter fluency.

### 3.3 Adjusted models

Results of all multilevel zero-inflated negative binomial models are presented in Table 3.

**Table 3:** Multilevel zero-inflated binomial models of engagement metrics.

Covariates	Total interactions (count)			Total duration (minutes)			Average interaction (minutes)		
	RR [95% CI]	*Z*	*p*	RR [95% CI]	*Z*	*p*	RR [95% CI]	*Z*	*p*
Unadjusted model fit	AIC = 19,910.00			AIC = 14,296.50			AIC = 5227.60		
Learn to Quit treatment arm^a^	**1.84** [1.27, 2.69]	**3.19**	**<0.01**	**8.43** [3.23, 21.99]	**4.36**	**<0.01**	**3.51** [1.89, 6.51]	**3.96**	**<0.01**

Adjusted model fit	AIC = 19,920.40			AIC = 14,302.20			AIC = 5233.50		
Learn to Quit treatment arm^a^	**1.72** [1.19, 2.49]	**2.85**	**<0.01**	**6.47** [2.59, 16.14]	**4.00**	**<0.01**	**2.70** [1.51, 4.81]	**3.35**	**<0.01**
Age	1.00 [0.98, 1.02]	0.32	0.75	1.01 [0.96, 1.05]	0.25	0.80	1.01 [0.98, 1.04]	0.70	0.49
Male sex^b^	**1.22** [1.01. 1.48]	**2.07**	**0.04**	0.90 [0.57, 1.42]	0.46	0.65	1.04 [0.78, 1.39]	0.29	0.77
Non-White^c^	1.46 [0.97, 2.21]	1.82	0.07	1.52 [0.56, 4.10]	0.82	0.41	1.28 [0.69, 2.38]	0.79	0.43
Post high school education^d^	1.11 [0.73, 1.67]	0.48	0.63	1.68 [0.62, 4.59]	1.02	0.31	1.49 [0.80, 2.79]	1.24	0.21
Schizophrenia spectrum diagnosis^e^	0.85 [0.54, 1.35]	0.69	0.49	0.82 [0.27, 2.46]	0.36	0.72	1.08 [0.55, 2.13]	0.22	0.83
Duration of illness	0.99 [0.98, 1.01]	0.53	0.59	1.02 [0.98, 1.06]	1.05	0.29	1.01 [0.98, 1.03]	0.70	0.48
PANSS	0.99 [0.97, 1.02]	0.67	0.50	1.05 [0.99, 1.12]	1.82	0.07	1.03 [0.99, 1.07]	1.87	0.06
BSI	1.12 [0.78, 1.60]	0.59	0.55	**0.37** [0.15, 0.91]	**2.16**	**0.03**	0.70 [0.40, 1.23]	1.24	0.22
AIS	1.01 [0.99, 1.03]	0.86	0.39	**1.09** [1.03, 1.15]	**2.99**	**<0.01**	**1.05** [1.02, 1.09]	**2.82**	**<0.01**
BACS	1.06 [0.89, 1.25]	0.62	0.53	1.47 [0.95, 2.28]	1.71	0.08	1.24 [0.95, 1.63]	1.56	0.12
False Belief Task	1.02 [0.98, 1.06]	0.98	0.33	1.02 [0.94, 1.12]	0.51	0.61	1.01 [0.96, 1.07]	0.35	0.72

Note: Bold values indicate *p* < .05, all variance inflation factor (VIF) values less than 1.05 suggesting multicollinearity is non-problematic.

AIS = Avoidance and Inflexibility Scale, BACS = Brief Assessment of Cognition in Schizophrenia, BSI = Brief Symptom Inventory. RR = Risk Ratio.

a QuitGuide is the reference group.

b Female is the reference group.

c Identifying as white racial identity is the reference group.

d Receiving a high school education or less is the reference group.

e Primary mood disorder diagnosis is the reference group.

### 3.4 Total interactions

**Fig. 2:**
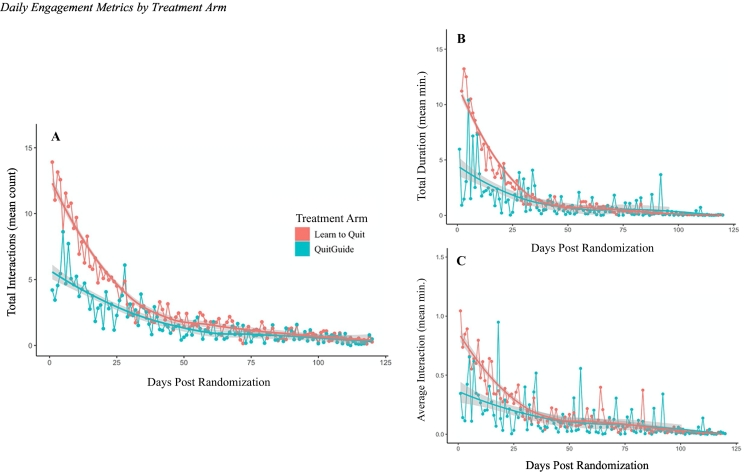

LTQ participants had significantly more interactions than QuitGuide (Risk Ratio (RR) [95% Confidence Interval] = 1.72 [1.19, 2.49], *Z* = 2.85, *p* < .01) with significant interindividual variability (*b* = 1.58, *Z* = 11.23, *p* < .01). Participant sex-assigned-at birth was also a significant predictor of daily interactions with male app users having more interactions (RR = 1.22 [1.01, 1.48], *Z* = 2.07, *p* = .04). See Fig. 2A for total daily interactions presented by treatment arm.

### 3.5 Total duration

LTQ participants interacted with the app for more minutes per day compared with QuitGuide participants (RR = 6.47 [2.59, 16.14], *Z* = 4.00, *p* < .01) with significant interindividual variability (*b* = 2.00, *Z* = 5.41, *p* < .01). Higher AIS scores were also a significant predictor of daily duration of app engagement (RR = 1.09 [1.03, 1.15], *Z* = 2.99, *p* < .01), while higher BSI global severity scores were a significant predictor of shorter duration of app engagement (RR = 0.37 [0.15, 0.91], *Z* = 2.16, *p* = .03). See Fig. 2B for average daily duration presented by treatment arm.

### 3.6 Average interaction

LTQ participants had significantly longer average interactions compared with QuitGuide participants (RR = 2.70 [1.51, 4.81], *Z* = 3.35, *p* < .01) with significant interindividual variability (*b* = 3.40, *Z* = 13.65, *p* < .01). Higher AIS scores were also a significant predictor of longer average app interaction (RR = 1.05 [1.02, 1.09], *Z* = 2.82, *p* < .01). See Fig. 2C for average daily interaction duration presented by treatment arm.

## 4 Discussion

This study examined the role of neurocognition, social cognition, and user characteristics in app engagement. Results showed that assignment to the tailored app (LTQ) remained the most robust predictor of app engagement across indices while adjusting for baseline, clinical, and cognitive characteristics. Results also demonstrated preliminary support for general symptom severity, male sex assigned at birth, and experiential avoidance as predictors of some indices of app engagement. Neurocognition and social cognition were not significantly associated with app engagement. However, lower cognitive functioning was *associated* with higher levels of engagement with the tailored app, suggesting digital therapeutics developed with SMI user input can engage a critically important segment of this population. This study found that most demographic and clinical characteristics, as well as social cognition and neurocognition ability, were not associated with engagement, suggesting that digital therapeutics can engage a broad and inclusive range of SMI users.

Findings echo results from an expert survey which identified app design elements (e.g., nonadherence to user-centered design principles) as critical barriers to engagement with digital interventions (Hatch et al., 2018). Findings from the present study are in line with previous work demonstrating that the implementation of user-centered design principles is a robust predictor of engagement, underscoring the need to use these methods to optimize digital health tools for SMI (Firth et al., 2016; Killikelly et al., 2017; Rotundi et al., 2007; Torous et al., 2018; Vilardaga et al., 2016). Interestingly, Torous et al. (2018) recommended against implementation of symptom tracking arguing that it may be tedious for some users. While LTQ and QuitGuide both include app interactions related to their symptom tracking feature, this study suggests that it is not simply the presence of a symptom tracking feature that may reduce engagement with a device, but how the symptom tracking feature is designed. This study is one of the first to demonstrate that use of a tailored app in SMI is associated with better engagement and related outcomes. Recent qualitative studies (e.g., Nicholson et al., 2017; Klein et al., 2019) demonstrate that there is an increasing interest in tailored apps for SMI users, and future work comparing the efficacy of these tailored apps may confirm findings from the present study.

Despite concerns that cognitive impairment may impact engagement in SMI users (e.g., Best et al., 2019; Thomas et al., 2018), model results suggest that SMI users with a broad range of neurocognitive abilities can meaningfully engage with apps, replicating previous findings from Luther et al. (2020) who found no associations between neurocognitive and social cognitive ability and engagement.

Results from correlation analyses demonstrated a striking pattern of associations between treatment arm and engagement. LTQ users with lower neurocognition and theory of mind ability had higher levels of engagement while no such relationships were observed in QuitGuide. These correlation analyses support treatment arm as a robust predictor of engagement in the adjusted models and suggests that use of a tailored app was especially effective in engaging SMI users with impaired social and neurocognition while still appealing to SMI users with intact cognition.

Results suggest that SMI users with higher experiential avoidance engaged for a longer duration of time with digital therapeutics. Higher experiential avoidance is transdiagnostically linked with greater psychopathology and with tobacco use disorder (Farris et al., 2015). Our results suggest that the LTQ app can meaningfully engage SMI users in treatment, despite the fact that the app encouraged users to make contact with uncomfortable smoking-related thoughts, feelings, and sensations, and that this could have triggered a strong avoidance response. In addition, users with more severe global psychiatric symptoms had less duration of engagement, whereas the same pattern was not observed among individuals with positive and negative symptoms. This finding is in line with previous equivocal studies that showed that severe symptoms predicted better app engagement in some studies but lower engagement in others (Buck et al., 2020; Granholm et al., 2012; Killikelly et al., 2017). Overall, these results reinforce the need to use design research (e.g., user-centered design) to address psychological factors from the target population (i.e., experiential avoidance, nicotine dependence, symptom severity) during the development of digital therapeutics.

Given the importance of engaging SMI users for optimal treatment outcomes in digital interventions, a recommendation based on the results of this study is made to conduct early-phase user-centered design research to directly address the potential impact of target sample specific factors (e.g., impaired cognition), on engagement with digital therapeutics. This study did not identify any robust demographic characteristics predictive of higher levels of engagement, which might suggest that LTQ engages a broad and inclusive range of users from this population. Therefore, another recommendation is to apply universal design principles when developing apps for SMI (Story, 1998). This approach may address key barriers to engagement and result in design features that are as inclusive as possible for the largest number of individuals within a population.

This study had limitations that should be considered when interpreting the results. Due to a software update, data on duration of app interaction is missing from eight participants in the QuitGuide group which reduced power. Examination of social cognition as a predictor was limited to theory of mind, which precludes generalizability to other domains of social cognition (e.g., social perception). Additionally, use of the BACS composite score limits generalizability to other neurocognition domains. Another limitation is the difference in opportunities to interact with app materials between treatment conditions. Although there was no maximum number of interactions, LTQ participants had more options than QuitGuide which may have increased engagement. However, impaired cognition, which has been associated with poor task performance (Bowie and Harvey, 2006), and distress tolerance (Chiappelli et al., 2014; Nugent et al., 2014), both present in SMI, could have led to frustration and to abandoning the modules offered by the LTQ app. Therefore, in the context of this population, more opportunities for interaction could have had the opposite effect of reduced levels of app engagement. Consistent with the hypothesis of previous user-centered design work (Vilardaga et al., 2018), results suggest that design features included in LTQ addressed these barriers which may have increased user engagement as defined across engagement outcomes. While participants were randomized to treatment conditions and consequently comparable across most baseline demographic and clinical characteristics, LTQ participants smoked significantly more cigarettes per day at baseline which may have increased treatment motivation and impacted engagement outcomes. Additionally, participants were able to retain ownership of a smartphone if they completed the study, and this may have increased treatment engagement across apps at a level higher than expected in a real-world setting. Finally, other unmeasured motivational factors could potentially contribute to the differential patterns of user engagement across apps.

This is the first study to evaluate neurocognitive and social cognitive predictors of app engagement by rigorously and directly measuring cognitive factors at the onset of a study. It is also the first study to evaluate the results of a research design effort to tailor a smoking cessation app to address these cognitive factors in the context of a non-tailored comparator. A significant strength of the study was the use of appropriate statistical models (e.g., zero-inflated distributions, interindividual variability) rather than aggregate approaches which can obfuscate patterns of prediction and contribute to equivocal findings.

In summary, LTQ's design appears to effectively predict engagement with a digital therapeutic among individuals with SMI after rigorously controlling for cognitive factors. As shown elsewhere (Browne et al., 2021), this finding is important since engagement with this digital therapeutic mediated clinical outcomes. Further, this tailored digital therapeutic seemed to comparably engage a broad range of individuals with different demographics and clinical characteristics from the target population. This study highlights the importance of user-centered design and provides preliminary support to address theory of mind and experiential avoidance to optimally engage individuals with SMI in the development of future digital therapeutics for this population.

## Role of funding sources

This study was funded by the 10.13039/100000026National Institute on Drug Abuse (R00 DA037276 and R01 DA047301 to R. Vilardaga). Dr. Browne is funded by the Department of Veterans Affairs Office of Academic Affiliations Advanced Fellowship in Geriatrics. Dr. Halverson is funded by the Department of Veterans Affairs Office of Academic Affiliations Mid-Atlantic Mental Illness Research, Education and Clinical Center. Statistical review was supported by the Duke Cancer Institute through 10.13039/100000002NIH grant P30CA014236 (PI: Kastan).

## Human rights

All procedures performed in studies involving human participants were in accordance with the ethical standards of the institutional and/or national research committee and with the 1964 Helsinki declaration and its later amendments or comparable ethical standards.

## Informed consent

Informed consent was obtained from all individual participants included in the study.

## Welfare of animals

This article does not contain any studies with animals performed by any of the authors.

## CRediT authorship contribution statement

**Tate F. Halverson:** Conceptualization, Methodology, Formal analysis, Writing – original draft, Writing – review & editing, Visualization. **Julia Browne:** Conceptualization, Writing – original draft, Writing – review & editing, Visualization. **Samantha M. Thomas:** Methodology, Resources, Writing – review & editing. **Paige Palenski:** Investigation, Writing – review & editing, Project administration. **Roger Vilardaga:** Conceptualization, Methodology, Investigation, Resources, Writing – original draft, Writing – review & editing, Data curation, Visualization, Supervision, Project administration, Funding acquisition.

## Declaration of competing interest

All authors declare that they have no conflicts of interest.

## Appendix A Supplementary data

Supplementary material Image 1
