# Supplementary material for: An examination of neurocognition and theory of mind as predictors of engagement with a tailored digital therapeutic in persons with serious mental illness
Source: Schizophr Res Cogn. 2022 Jan 17;28:100236. doi: 10.1016/j.scog.2022.100236 (PMC8861409; doi:10.1016/j.scog.2022.100236)
Supplement: Supplementary file 1 — Supplementary material [file mmc1.docx]

Supplementary Materials

Supplementary Table 1

*Correlation between Predictors by Treatment Group*

|  |  | Demographics | | | | Clinical Characteristics | | | | | | | Cognitive Performance | | | | | | | | |
| --- | --- | --- | --- | --- | --- | --- | --- | --- | --- | --- | --- | --- | --- | --- | --- | --- | --- | --- | --- | --- | --- |
|  |  | 1 | 2 | 3 | 4 | 5 | 6 | 7 | 8 | 9 | 10 | 11 | 12 | 13 | 14 | 15 | 16 | 17 | 18 | 19 | 20 |
| Demographics | 1. Age | - | .13 | .22 | .35 | .23 | .26 | -.46 | .27 | -.22 | -.13 | .05 | -.17 | -.27 | -.47 | -.13 | -.20 | -.20 | -.49 | -.42 | -.20 |
|  | 2. Male | -.52 | - | .28 | .46 | .25 | -.16 | -.01 | -.21 | .03 | -.35 | -.32 | -.13 | -.07 | -.40 | -.06 | .00 | -.09 | -.11 | -.36 | -.10 |
|  | 3. Education | -.14 | .00 | - | .09 | .65 | -.21 | -.23 | -.26 | .14 | .01 | .06 | -.08 | -.31 | -.20 | -.12 | .18 | .11 | -.15 | -.23 | -.13 |
|  | 4. Race^a^ | -.09 | -.04 | .21 | - | .25 | .06 | -.26 | .19 | .39 | -.13 | -.14 | **-.84** | -.56 | -.70 | -.75 | -.65 | **-.83** | -.51 | -.65 | -.44 |
| Clinical Characteristics | 5. SSD Diagnosis | -.05 | .30 | .12 | .40 | - | - | - | -.04 | .85 | .43 | .31 | -.49 | -.16 | -.54 | -.53 | -.25 | -.19 | -.57 | -.60 | -.79 |
|  | 6. Bipolar Diagnosis | .04 | -.09 | .00 | -.66 | - | - | - | .03 | -.44 | -.11 | .18 | .31 | .10 | .10 | .37 | .03 | .08 | .25 | .36 | .11 |
|  | 7. Major Depressive Diagnosis | .01 | -.24 | -.14 | .45 | - | - | - | -.01 | -.12 | -.19 | -.43 | .01 | .00 | .28 | -.03 | .15 | .05 | .13 | .03 | .44 |
|  | 8. Duration of Treatment | .55 | -.27 | -.28 | .11 | -.05 | .08 | -.04 | - | -.04 | -.20 | -.31 | -.14 | -.05 | -.26 | -.16 | -.21 | -.15 | -.03 | -.11 | -.05 |
|  | 9. PANSS - Total | .16 | .09 | -.24 | -.05 | -.02 | .21 | -.25 | .19 | - | .58 | .08 | -.24 | .05 | -.13 | -.29 | -.14 | -.19 | -.19 | -.18 | -.37 |
|  | 10. BSI – Global Severity | .10 | .11 | -.22 | .13 | .00 | .00 | .01 | .08 | **.66** | - | .11 | .03 | .08 | .12 | .05 | .06 | -.06 | -.14 | .21 | -.14 |
|  | 11. ASI - Total | .11 | .02 | .13 | -.04 | -.08 | .23 | -.20 | .05 | .33 | .45 | - | .02 | -.24 | .27 | .06 | -.06 | -.05 | .00 | .08 | -.23 |
| Cognitive Performance | 12. BACS – Composite | -.09 | .12 | -.37 | -.44 | -.51 | .22 | .29 | -.04 | -.23 | -.16 | -.23 | - | **.63** | **.69** | **.84** | **.63** | **.79** | .43 | **.72** | .42 |
|  | 13. BACS – Verbal Memory | -.29 | .04 | .01 | -.19 | -.29 | -.04 | .37 | -.17 | -.19 | .00 | -.02 | **.71** | - | .30 | .30 | .40 | .57 | .18 | .33 | .11 |
|  | 14. BACS – Processing Speed | -.49 | .13 | -.27 | -.08 | -.43 | .08 | .38 | -.29 | -.47 | -.29 | -.31 | **.74** | .53 | - | .55 | .42 | .51 | .59 | **.66** | .30 |
|  | 15. BACS – Working Memory | -.03 | .20 | -.33 | -.39 | -.29 | .28 | -.03 | -.05 | -.14 | -.13 | -.13 | **.70** | .32 | .55 | - | .62 | .62 | .44 | **.65** | .58 |
|  | 16. BACS – Verbal Fluency - S | -.05 | .14 | -.23 | -.18 | -.38 | .33 | .00 | .00 | -.07 | .17 | -.08 | **.65** | .44 | .47 | .40 | - | **.73** | .41 | .26 | .54 |
|  | 17. BACS – Verbal Fluency - L | -.13 | .14 | -.38 | -.20 | -.25 | .23 | .00 | .01 | -.25 | -.28 | -.31 | **.75** | .37 | .59 | .52 | .58 | - | .39 | .32 | .37 |
|  | 18. BACS – Motor Function | -.38 | .15 | -.23 | .02 | -.32 | -.04 | .40 | -.07 | -.33 | -.23 | -.25 | .56 | .48 | **.68** | .58 | .15 | .40 | - | .46 | .56 |
|  | 19. BACS – Executive Function | -.27 | .10 | -.04 | -.52 | -.44 | .20 | .23 | -.19 | -.12 | -.13 | -.19 | **.67** | .48 | .49 | .31 | .41 | .44 | .29 | - | .35 |
|  | 20. False Belief Task | -.33 | .06 | .19 | -.12 | -.07 | .26 | -.25 | -.29 | -.13 | .02 | -.10 | .35 | .51 | .41 | .25 | .42 | .14 | .20 | .30 | - |

*Note*: Learn to Quit correlations presented below the diagonal, QuitGuide correlations presented in grey above the diagonal. Bold values indicate *p* <.0025 the adjusted significance for multiple comparisons using Holm-Bonferroni correction; SSD = schizophrenia spectrum disorder; PANSS = Positive and Negative Syndrome Scale, BSI = Brief Symptom Inventory, AIS = Avoidance and Inflexibility Scale, BACS = Brief Assessment of Cognition in Schizophrenia, BACS – Verbal Fluency – S = semantic fluency, BACS – Verbal Fluency – L = letter fluency.

Supplementary Figure 1

*Histograms of App Engagement Metrics*

**

*Note*: Top row includes all data, bottom row presents enlarged histograms without zero counts.
